# Effect of Zearalenone-Induced Ferroptosis on Mice Spermatogenesis

**DOI:** 10.3390/ani12213026

**Published:** 2022-11-03

**Authors:** Yajing Li, Zhendong Zhu, Haixiang Cui, Kexin Ding, Yong Zhao, Xiangping Ma, Adedeji Olufemi Adetunji, Lingjiang Min

**Affiliations:** 1 College of Animal Science and Technology, Qingdao Agricultural University, Qingdao 266109, China; 2 State Key Laboratory of Animal Nutrition, Institute of Animal Sciences, Chinese Academy of Agricultural Sciences, Beijing 100193, China; 3 Department of Animal Sciences, North Carolina Agricultural and Technical State University, Greensboro, NC 27411, USA

**Keywords:** zearalenone (ZEA), ferroptosis, system Xc^−^, spermatogenesis, male reproductive

## Abstract

**Simple Summary:**

Zearalenone is a mycotoxin that can cause reproductive toxicity after long-term feeding in domestic animals and it affects spermatogenesis in male domestic animals. Ferroptosis is a newly identified type of programmed cell death, which depends on iron accumulation and lipid peroxidation. Fer-1 inhibits the process of ferroptosis. However, it is not clear whether ferroptosis plays a role in zearalenone (ZEA) damage to spermatogenesis. This study establishes a ZEA damage model of in male mice. After Fer-1 intervention, it was found that Fer-1 improves the antioxidant system of mice testis, reduces iron levels, restores related factors of *Nrf2*, *SLC7A11*, and *GPX4* to normal levels, and accelerates reproductive injury recovery.

**Abstract:**

Male reproductive health is critically worsening around the world. It has been reported that the mycotoxin ZEA causes reproductive toxicity to domestic animals and affects spermatogenesis, thereby inhibiting male reproductive function. Ferroptosis is a newly identified type of programmed cell death that is different from apoptosis and it depends on iron accumulation and lipid peroxidation. Whether ferroptosis is linked to ZEA’s detrimental effect on spermatogenesis needs to be further explored. This study clarifies ferroptosis’s involvement in ZEA-induced damage on spermatogenesis. The reproductive injury model used in this study was induced by gavaging male mice in the ZEA treatment group with 30 μg/kg of ZEA for five weeks. Results show that ZEA treatment reduced mouse sperm motility and concentration, destroyed the structure of the seminiferous tubules of the testis, damaged the antioxidant defense system, and blocked spermatogenesis. Ferrostatin-1 (Fer-1) inhibition of ferroptosis partially alleviated ZEA-induced oligozoospermia in mice. In addition, ZEA treatment was found to activate a signaling pathway associated with ferroptosis in mouse testis. ZEA also downregulated the expression of *Nrf2*, *SLC7A11*, and *GPX4*, and decreased the protein expression of SLC7A11 and GPX4, resulting in the accumulation of lipid peroxides and an increase in the level of 4-HNE protein in the testis. Importantly, these changes were accompanied by an increase in the relative contents of Fe^2+^ and Fe^3+^. Iron accumulation and lipid peroxidation are the causes of ferroptosis in spermatogenic cells, leading to a decrease in sperm motility and concentration. While the administration of Fer-1 at 0.5 and 1 mg/kg also increased the expression of SLC7A11 and GPX4 proteins by upregulating *Nrf2* expression, reducing iron accumulation, and reversing ZEA-induced ferroptosis, Fer-1 at 1.5 mg/kg had the best repairing effect for all parameters. In conclusion, ZEA-induced ferroptosis may be mediated by a notable reduction in *Nrf2*, *SLC7A11* and *GPX4* expression levels. Overall, ferroptosis is a novel therapeutic target for mitigating ZEA-induced reproductive toxicity.

## 1. Introduction

Zearalenone (ZEA) is an estrogen-like mycotoxin produced by fusarium fungi, which is widely distributed in wheat [1], corn [2], soybean [3], and other crops. It affects meat and dairy products consumed by humans thus posing a serious threat to animal and human health [4,5]. Previous studies showed that ZEA not only causes cytotoxicity [6], genetic toxicity [7], and immunotoxicity [8] in domestic animals but also affects the normal operation of the estrogen signaling pathway leading to animal reproductive toxicity [9]. Previous studies have shown that ZEA exposure can reduce the semen quality of male mice, increase the rate of spermatogenic cell apoptosis, hinder spermatogenesis, and thus impair male reproductive function [10,11]. 

Ferroptosis is an iron-dependent non-apoptotic form of programmed cell death characterized by iron-dependent accumulation of lipid peroxides [12]. Moreover, ferroptosis is regulated by a variety of cellular metabolic pathways, including redox homeostasis, iron processing, mitochondrial activity, and the metabolism of amino acids, lipids, and sugars [13,14,15].

Glutathione peroxidase 4 (GPX4), a key factor in ferroptosis, is a negative regulator of ferroptosis [16], and the most important anti-lipid peroxidase in cells. GPX4 converts lipid peroxides into non-toxic alcohols under the synergistic action of reduced glutathione (GSH) [17,18]. Therefore, GSH is also an important cofactor of GPX4, and the depletion of GSH will lead to the inactivation of *GPX4* and affect its activity, thereby increasing intracellular lipid peroxidation and leading to ferroptosis [19]. Importantly, cystine, the raw material for GSH synthesis, is taken up extracellularly via system Xc^−^, which is mainly composed of SLC7A11 [20,21]. Studies have shown that nuclear factor erythroid-2-related factor 2 (Nrf2) is a key factor regulating antioxidant activity and can regulate the levels of SLC7A11 [22]. Therefore, inhibitions of system Xc^−^ and GPX4 activity are important conditions for ferroptosis.

Importantly, Nrf2, SLC7A11, and GPX4 are closely related to spermatogenesis. The loss of GPX4 in mouse spermatocytes leads to a significant decrease in sperm concentration and motility [23]. Clinical studies have shown that decreased *Nrf2* expression is closely related to oligozoospermia [24]. In addition, inhibition of the cysteine transporter SLC7A11 in the system Xc^−^ also decreased sperm motility [25]. Therefore, given the importance of Nrf2, SLC7A11, and GPX4 in ferroptosis and spermatogenesis, we investigated ferroptosis’s involvement in the spermatogenesis process.

In this study, the effect of ZEA on spermatogenesis and changes in antioxidant indexes in male mice were explored. At the same time, the relative contents of Fe^2+^ and Fe^3+^ as well as changes in *Nrf2*, *SLC7A11*, and *GPX4* expression levels were detected in mouse testes when ferrostatin-1 (Fer-1) was administered with ZEA. Likewise, the correlation between ferroptosis and ZEA-induced male reproductive injury was further demonstrated.

## 2. Materials and Methods

### 2.1. Materials

Routine chemicals and reagents were purchased from Solarbio (Shanghai Solarbio Bioscience & Technology Co. Ltd., Shanghai, China) or Sigma Chemical Co. (St. Louis, MO, USA).

### 2.2. Animal Processing

The animal study protocol was approved by the Qingdao Agriculture University Institutional Animal Care and Use Committee (Ethics Approval Code: QAU202011). Three-week-old ICR male mice were used in this study and they were divided into vehicle control and treatment groups. The control group was subjected to daily gavage with saline while the treatment group was gavaged daily with ZEA for a total of 5 weeks. At four weeks of age, mice were injected with different concentrations of Fer-1 (0.5, 1, 1.5 mg/kg). In total, there were five groups (*n* = 10 per group): (1) Con-sa (dosed with saline); (2) ZEA (ZEA (30 μg/kg BW of ZEA) plus saline); (3) ZEA + Fer-1 (ZEA plus Fer-1 (0.5 mg/kg)); (4) ZEA + Fer-1 (ZEA plus Fer-1 (1 mg/kg)); (5) ZEA + Fer-1 (ZEA plus Fer-1 (1.5 mg/kg)). Mice were maintained under a light: dark cycle of 12:12 h at a temperature of 23 °C and humidity of 50–70% with free access to food (chow diet) and water [26]. 

### 2.3. Animal Weight and Testicular Weight

The body weight of mice in each treatment group was weighed before euthanasia, then both testicles were dissected and weighed.

### 2.4. Spermatozoa Motility Determined by a Computer-Assisted Sperm Analysis System

Spermatozoa motility was assessed using a computer-assisted sperm assay (CASA) method according to World Health Organization guidelines [27]. After euthanasia, spermatozoa were collected from the cauda epididymis of mice and suspended in DMEM/F12 medium with FBS (*v*:*v* = 9:1, final concentrations: 10%) and incubated at 37.5 °C for 30 min; 5 μL of diluted semen from each treatment group was collected and placed in a pre-warmed counting chamber (SCA 20-06-01; Goldcyto, Barcelona, Spain), then sperm concentrations and motility were analyzed by the CASA system. The microscopic sperm class analyzer (CASA system) was used in this investigation. It was equipped with a 20-fold objective, a camera adaptor (Eclipse E200, Nikon, Japan), and a camera (acA780-75gc, Basler, Germany). More so, it was operated by an SCA sperm class analyzer (MICROPTIC S.L.). Then, indexes including sperm concentration, motility, and other parameters were comprehensively assessed. The classification of sperm motility was as follows: grade A = linear velocity > 22 μm/s; grade B = linear velocity < 22 μm/s and curvilinear velocity > 5 μm/s; grade C = curvilinear velocity < 5 μm/s; and grade D = immotile spermatozoa. Sperm concentration and motility were evaluated and diluted with 10% FBS medium during the process of evaluation.

### 2.5. Hematoxylin and Eosin Staining

Mouse testes were collected and fixed in 4% paraformaldehyde, stored overnight in a refrigerator at 4 °C, and then incubated in different concentrations of dehydrating solutions. The dehydrated testicular samples were then embedded in paraffin and the resulting paraffin blocks were sectioned at a thickness of 5 μm according to standard histological procedures. Testicular sections were stained with hematoxylin and eosin staining.

### 2.6. Measurement of MDA, T-GSH Contents

The content of MDA and T-GSH in testicular tissue was detected using commercial kits (A003-1-2; A061-1-1; Nanjing Jiancheng Bioengineering Institute) according to the manufacturer’s regulations. The testes tissues and saline (1:9) were homogenized and centrifuged at 10,000× *g* for 10 min, the precipitate was discarded and the supernatant was collected. Then, the supernatant was mixed with the assay buffer and substrates, and measured at 532 nm and 405 nm with a micro-plate reader for MDA and T-GSH levels, respectively. Analyses were performed in triplicate (*n* ≥ 3); the cv values are shown in Supplementary Table S1.

### 2.7. Evaluation of SOD Activity

The total superoxide dismutase assay kit (A001-3-2; Nanjing Jiancheng Bioengineering Institute) was used to assess the activity of superoxide dismutase (SOD). Testicular homogenate was centrifuged at 10,000× *g* for 10 min at 4 °C to collect the supernatant. The protein concentrations were quantified using the bicinchoninic acid method. Then, the supernatant was mixed with the assay buffer and the SOD activity was calculated according to the absorbance at 450 nm. Analyses were performed in triplicate (*n* ≥ 3); the cv values are shown in Supplementary Table S1.

### 2.8. Measurement of Fe^3+^ and Fe^2+^ Contents

The content of Fe^3+^ and Fe^2+^ in testicular tissues was detected using an iron colorimetric assay kit (E-BC-K139-S, Elabscience, Wuhan, China) and ferrous iron colorimetric assay kit (E-BC-K773-M, Elabscience, Wuhan, China) according to the manufacturer’s instructions. Testicular tissues were taken and rapidly placed in ice-cold saline to prepare tissue homogenates, then centrifuged at 12,000× *g* for 10 min at 4 °C. The supernatant obtained was used to measure various indicators. The relative content level of Fe^3+^ and Fe^2+^ was calculated and normalized to mean control levels. Analyses were performed in triplicate (*n* ≥ 3); the cv values are shown in Supplementary Table S1.

### 2.9. Quantitative Real-Time PCR Assay

Total RNA was extracted from testicular tissues using an RNA extraction kit (Sparkjade Biotechnology Co., LTD, Shandong, China). Then, 2 μg of RNA was reverse transcribed using HiScript Ⅱ1 s Strand cDNA Synthesis Kit (+gDNA wiper) obtained from Vazyme Biotech Co., Ltd. Briefly, 2 μg of total RNA was used to make the first strand of complementary DNA (cDNA; in 20 μL) using an RT2 First Strand Kit (Cat. No: AT311-03, Transgen Biotech, Beijing, China) following the manufacturer’s instructions. The generated first-strand cDNAs (20 μL) were diluted to 150 μL with double-deionized water (ddH_2_O). Then, 1 μL was used for one PCR reaction (in a 96-well plate). Each PCR reaction (12 μL) contained 6 μL of qPCR Master Mix (Roche, Basel, Switzerland), 1 μL of diluted first strand cDNA, 0.6 μL primers (10 mM), and 4.4 μL of ddH2O. The primers for qPCR analysis were synthesized by Tsingke. qPCR was conducted by using a Roche LightCycler 480 (Roche, Basel, Switzerland) with the following program: step 1: 95 °C, 10 min; step 2: 40 cycles of 95 °C, 15 s; 60 °C, 1 min; step 3: dissociation curve; step 4: cool down; *n* = 3/group. Additionally, the relative abundance of mRNA was calculated and normalized to mean β-actin mRNA levels. The primer sequences used in this study are presented in Table 1.

### 2.10. Western Blotting (WB)

WB analysis was carried out as described in our previous report [28]. Proteins from testes tissue were lysed in RIPA buffer containing a protease inhibitor mixture. A BCA protein concentration kit was used to determine the concentration of extracted protein, and the concentration was unified. An equal volume of total protein was separated on SDS-PAGE gels. The proteins were then transferred onto PVDF membranes (GE Bioscience, Newark, NJ, USA) and then blocked with 5% BSA (dissolved in TBST) for 1 h and the membrane was incubated with anti-SYCP3 (ab97672; Abcam, Cambridge, UK), anti-DDX4 (bs-22896R; Bioss Biotech, Beijing, China), anti-SOX9 (ab185966; Abcam, Cambridge, UK), anti-SLC7A11 (bs-6883R; Bioss Biotech, Beijing, China), anti-GPX4 (bs-3884R; Bioss Biotech, Beijing, China), anti-4-HNE (ab48506; Abcam), and anti-β-actin (bs-0061R; Bioss Biotech, Beijing, China) overnight at 4 °C. The next day, the PVDF membrane was washed with TBST and incubated with a secondary antibody (1:1000 in TBST) for 1 h at room temperature. Finally, the bands were quantified using Image J software. The intensity of specific protein bands was normalized to β-actin.

### 2.11. Statistical Analyses

All data were first tested for normality and variance homogeneity through the Shapiro–Wilk and Levene tests, respectively. When necessary, data were transformed by arc-sin square root transformation prior to statistical analysis. All the values are presented as the mean ± SEM. The data were analyzed by SPSS statistical software (SPSS, Inc., Chicago, IL, USA) using a one-way analysis of variance (ANOVA) followed by the LSD multiple comparisons test. Graphs were created using GraphPad Prism 5.20 (GraphPad Software Inc., La Jolla, CA, USA). All groups were compared with each other for every parameter. *p* < 0.05 was considered statistically different from one another.

## 3. Results

### 3.1. Fer-1 Protects against ZEA-Induced Reduction of Body Weight and Sperm Motility

**Figure 1:**
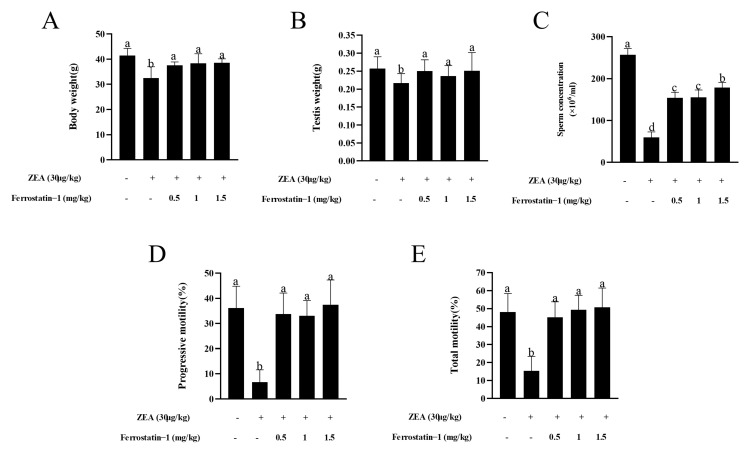
ZEA induced a reduction of body weight, testis weight, sperm concentration, and sperm motility in mice that was alleviated by administration of Fer-1. (**A**) Body weight of all groups of mice is presented as a bar graph. (**B**) Testis weight of all groups of mice is presented as a bar graph. (**C**) Epididymal sperm concentrations in experimental mice are presented as a bar graph. (**D**) Epididymal progressive sperm motility in experimental mice is presented as a bar graph. (**E**) Epididymal total sperm motility in mice is presented as a bar graph. The data are expressed as means ± SEM, with *n* > 6 per group; a, b, c, d means not sharing a common superscript are different (*p* < 0.05).

As shown in Figure 1, ZEA treatment resulted in a significant reduction in body weight, testis weight, sperm concentration, and total motility, as well as progressive motility when compared to the treatment without ZEA or Fer-1 (*p* < 0.05). In addition, administration of the ferroptosis inhibitor Fer-1 from 0.5 mg/kg to 1.5 mg/kg significantly increased the body weight, testis weight, sperm concentration, total motility, and progressive motility compared to the ZEA treatment (*p* < 0.05) (Figure 1A–E). When the sperm motility patterns were analyzed, it was observed that the ZEA treatment decreased the VSL, VCL, VAP, LIN, STR, and WOB parameters; meanwhile, the addition of Fer-1 from 0.5 mg/kg to 1.5 mg/kg alleviated the damage induced by ZEA for those parameters (*p* < 0.05) (Table 2).

### 3.2. Fer-1 Can Alleviate ZEA-Induced Pathological Changes in Mouse Testis and Increases the Expression of Important Genes Involved in Spermatogenesis

**Figure 2:**
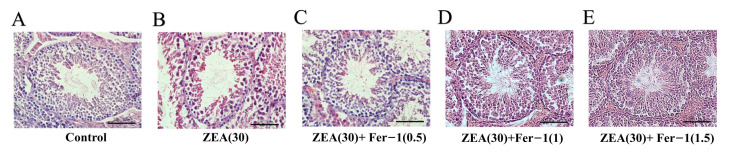
Seminiferous tubule architecture in hematoxylin eosin (H&E) stained sections in the testes of experimental mice. (Scale bar = 20 μm).

The changes in testicular tissue were observed at the end of the five week treatment with ZEA by HE staining. The results showed that, in the control group, the diameters of seminiferous tubules were larger (Supplementary Figure S1), and various spermatogenic cell types in the testicular tissue were complete and orderly (Figure 2A). Meanwhile, in ZEA group, the conformation of the tubules changed, the diameters of seminiferous tubules decreased (Supplementary Figure S1), the cells were not closely arranged, and some vacuoles were observed in the tubules (Figure 2B). In addition, after treatment with the ferroptosis inhibitor Fer-1 from 0.5 mg/kg to 1.5 mg/kg, the results showed that germ cells in the spermatogenic tubules were restored to a certain extent, the cells were orderly arranged, and the testicular structure and germ cells were similar to the blank control group (Figure 2C–E).

**Figure 3:**
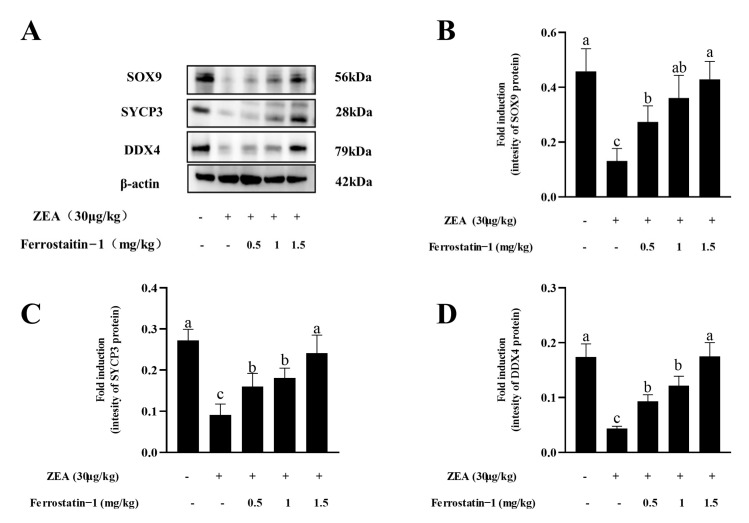
Expressions of SOX9, SYCP3, and DDX4 in the testis of mice in each group. (**A**) Western blot detection of SOX9, SYCP3 and DDX4 protein. (**B**) Image J analysis showing the grey value of SOX9. (**C**) Image J analysis showing the grey value of SYCP3. (**D**) Image J analysis showing the grey value of DDX4. The data are expressed as means ± SEM, with *n* ≥ 3; Different lowercase letters (a, b, c) indicate significant differences (*p* < 0.05).

*SYCP3*, *DDX4* and *SOX9* are markers of spermatocytes, male germ cells and Sertoli cells, respectively. It was observed that the ZEA treatment significantly decreased the levels of SYCP3, DDX4 and SOX9 proteins in the testis, while the levels of those proteins were increased by the treatment with the ferroptosis inhibitor Fer-1 from 0.5 mg/kg to 1.5 mg/kg (Figure 3A–D, Supplementary Figure S2). 

### 3.3. Fer-1 Can Affect the Contents of MDA, T-GSH, SOD Activity, Fe^3+^ and Fe^2+^ in Mouse Testis

**Figure 4:**
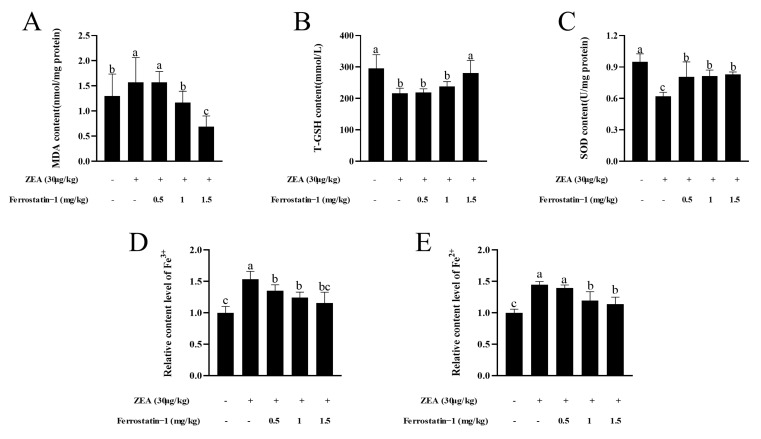
Oxidation indicators and iron detection related to ferroptosis. (**A**) MDA content. (**B**) Total GSH content. (**C**) SOD content. (**D**) Relative content level of Fe^3+^. (**E**) Relative content level of Fe^2+^. The data are expressed as means ± SEM, *n* ≥ 3; Different lowercase letters (a, b, c) indicate significant differences (*p* < 0.05).

fTo determine whether ZEA exposure causes a change in the oxidation index of testicular tissue, the content of malondialdehyde (MDA) in the testis was examined. Results showed that ZEA exposure significantly increased MDA content in the testis (*p* < 0.05). Meanwhile, the contents of T-GSH and SOD activity in ZEA treatment group were fewer than those in the control group (*p* < 0.05) (Figure 4A–C). After Fer-1 treatment, the MDA, and T-GSH contents and SOD activity increased gradually with increasing Fer-1 concentrations, and the rescue effect of Fer-1 was more obvious at a concentration of 1.5 mg/kg (*p* < 0.05) (Figure 4A–C). As shown in Figure 4D,E, the relative levels of Fe^3+^ and Fe^2+^ in ZEA treatment group were higher than those in the control group (*p* < 0.05). After the injection of Fer-1, the level of the elements was reduced (*p* < 0.05).

### 3.4. Fer-1 Can Alter ZEA-Induced Ferroptosis-Related Gene and Protein Levels

**Figure 5:**
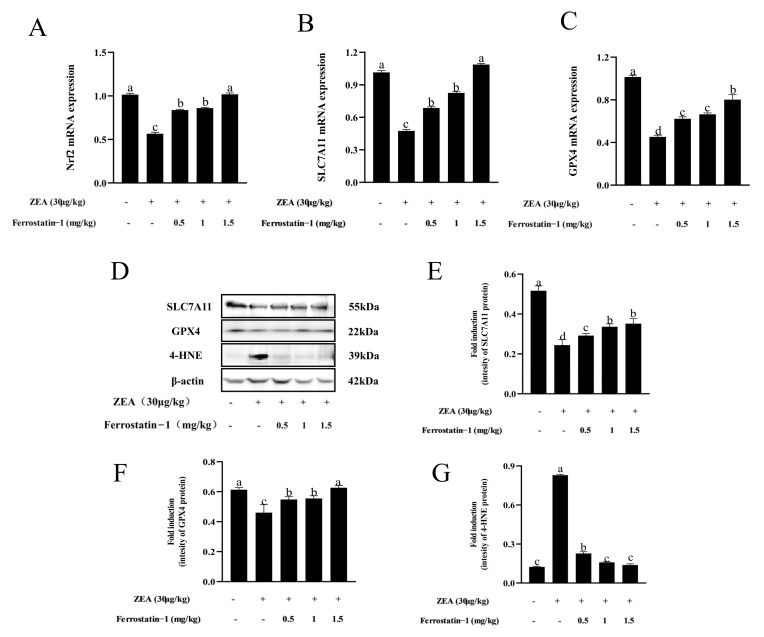
Expressions of genes, proteins involved in ferroptosis in testis of mice in each group. (**A**) *Nrf2* mRNA expression. (**B**) SLC7A11 mRNA expression. (**C**) GPX4 mRNA expression. (**D**) Western blot detection of SLC7A11, GPX4, and 4-HNE protein. (**E**) Image J analysis showing the grey value of SLC7A11. (**F**) Image J analysis showing the grey value of GPX4. (**G**) Image J analysis showing the grey value of 4-HNE. The data are expressed as means ± SEM, with *n* ≥ 3; Different lowercase letters (a, b, c, d) indicate significant differences (*p* < 0.05).

As shown in Figure 5A–C, when compared to the control group, the expression of *Nrf2*, *SLC7A11*, and *GPX4* were down-regulated in the ZEA group; meanwhile, the addition of Fer-1 increased the *Nrf2*, *SLC7A11*, and *GPX4* expression in the testis (*p* < 0.05) (Figure 5A–C). Interestingly, *SLC7A11* and *GPX4* protein expressions were decreased, whereas 4-HNE protein expression significantly increased in ZEA treatment (*p* < 0.05) (Figure 5D–G). After Fer-1 intervention, the expression of *SLC7A11* and *GPX4*-related proteins increased, while the expression of 4-HNE protein decreased (Figure 5D–G, Supplementary Figure S3).

## 4. Discussion

Previous studies have reported that zearalenone (ZEA) is one of the mycotoxins that exist in contaminated food [29]. Zearalenone has been found to impair male reproduction, reducing semen quality and impeding spermatogenesis [7,30]. The results of this study showed that after 5 weeks of ZEA (30 μg/kg) treatment, the ZEA treatment group significantly reduced the sperm motility, concentration, and sperm motility parameters of mice. *SOX9* is expressed only in the Sertoli cells of the testis and plays an important role in testicular development [31,32]. Spermatogenesis is regulated by *SYCP3* and *DDX4*. It was found that ZEA exposure changed the testicular structure and reduced the expression of SOX9, SYCP3, and DDX4 proteins in testes, indicating that ZEA hindered testicular development and spermatogenesis by destroying proteins involved in spermatogenesis.

Environmental toxins can induce oxidative stress to produce reproductive toxicity [33]. Studies have shown that ZEA can induce oxidative damage, which may be one of the main pathways of ZEA toxicity [34,35]. Our results, which show that ZEA treatment increased MDA content, decreased T-GSH content and SOD activity in testicular tissue, are consistent with the results of previous studies [33]. This suggests that ZEA inhibits spermatogenesis and reduces the antioxidant capacity of testicular tissue by reducing the effects of key antioxidant enzymes. However, the mechanism by which ZEA affects spermatogenesis remains unclear and needs to be further studied.

Ferroptosis is a new form of cell death, which plays a role in various diseases, including cancer cell death [36], neurodegenerative disease [37], heart ischemia/reperfusion injury [38], and other life processes. Ferroptosis mainly depends on iron accumulation and lipid peroxidation [39]. It was confirmed that ZEA could induce lipid peroxidation in testicular tissue, but surprisingly, the relative content levels of Fe^3+^ and Fe^2+^ in testicular tissue were increased after ZEA treatment compared with the control group, suggesting that ZEA induces lipid peroxidation and iron accumulation in testicular tissue, which may cause ferroptosis. Therefore, it was speculated that ZEA may affect the process of male spermatogenesis through ferroptosis. Interestingly, supplementation of 0.5 to 1.5 mg/kg Fer-1 alleviated ZEA-induced testicular damage, inhibited iron levels, and increased sperm motility and sperm concentration, with 1.5 mg/kg being the most effective. 

Nrf2 is an important antioxidant transcription factor that regulates the expression of some cytoprotective genes by binding its reactive elements, which are involved in detoxification and antioxidant and drug metabolism [40]. Many ferroptosis-related genes are transcriptionally regulated by *Nrf2*, which negatively regulates ferroptosis [41]. Notably, ZEA inhibits the expression of *Nrf2* in porcine testicular cells and reduces the antioxidant capacity of cells [42]. More so, the deletion of *Nrf2* gene in male mice causes a decrease in sperm density and motility rate [43]. In view of the wide applications of *Nrf2* in ferroptosis and reproduction, the mRNA expression level of *Nrf2* was detected in this study. It was found that exposure to ZEA reduced the expression of *Nrf2*. Interestingly, the mRNA expression level of *Nrf2* increased after different concentrations of Fer-1 was administered, indicating that *Nrf2* is involved in ZEA-induced ferroptosis.

SLC7A11, one of the components of the system Xc^−^, is a major signaling pathway associated with ferroptosis, is involved in the extracellular to intracellular uptake of cystine and determines the synthesis of glutathione (GSH) [44]. Interestingly, studies have shown that SLC7A11 is a transcriptional target of Nrf2 [45,46]. We explored the relationship between ZEA exposure and SLC7A11 expression. Results showed that the protein expression and mRNA content of SLC7A11 in the ZEA treatment group were significantly lower than those in the control group, and the protein and gene levels of SLC7A11 gradually increased after 0.5 mg/kg to 1.5 mg/kg of Fer-1 was administered. Therefore, it was speculated that ZEA inhibited the expression of SLC7A11 in mouse testis and inhibited system Xc^−^ function, which may be involved in *Nrf2*-mediated ferroptosis.

GPX4 is considered to be a central regulator of ferroptosis. GPX4 is an enzyme that reduces lipid hydrogen peroxide to non-toxic lipid alcohols and induces ferroptosis when its activity is inhibited. Moreover, when the system Xc^−^ function is inhibited, the inhibition of GSH synthesis also affects the activity and expression of GPX4, thereby regulating ferroptosis [44]. Interestingly, clinical studies have shown that the failure of sperm mitochondrial PHGPx expression may be one of the causes of oligoasthenospermia in infertile men [47]. In addition, when GPX4 was specifically lost in mice, the number of sperm cells in the epididymis was reduced, resulting in impaired sperm quality [23]. GPX4 plays an important role in ferroptosis and spermatogenesis. As mentioned in the previous results, T-GSH content in mouse testis decreased due to ZEA exposure. We also found that ZEA exposure decreased GPX4 protein and mRNA expression, until the intervention of 0.5 to 1.5 mg/kg of Fer-1. This demonstrates that GPX4 expression decreased when the expression of GSH decreased, a condition that favors the occurrence of ferroptosis.

One of the characteristics of ferroptosis is lipid peroxidation. In this study, the changes of 4-HNE protein level were detected at the molecular level. 4-HNE belongs to the advanced lipid peroxidation end product family [48], which is involved in protein dysfunction, apoptosis, inflammatory damage, and other cytotoxic processes [49,50,51]. It can be used as a biomarker of lipid peroxidation and oxidative stress [48,52]. Our results show that the ZEA treatment group significantly increased the expression of 4-HNE protein in testicular tissue, indicating that the lipid peroxides in testicular tissue significantly increased. Intriguingly, after Fer-1 intervention, the level of 4-HNE protein was restored to normal levels as observed in the control group.

## 5. Conclusions

In conclusion, ZEA may inhibit the system Xc^−^ function of downstream proteins SLC7A11 and GPX4 by down-regulating the expression of *Nrf2*, causing iron accumulation and lipid peroxidation, and inducing ferroptosis in mice testes. Eventually, ZEA administration resulted in reduced sperm motility and concentration and hindered spermatogenesis. Overall, our results suggest that ferroptosis plays an important role in ZEA-induced oligozoospermia.

## Figures and Tables

**Table 1:** List of primers employed for q-RT-PCR.

Gene Name	Primer Sequence
*Nrf2*	F:5′-ATGACTCTGACTCTGGCATTTC-3′
	R:5′-GCACTATCTAGCTCCTCCATTTC-3′
*SLC7A11*	F:5′-GTGGGAGGCTGGTAGTTAATG-3′
	R:5′-CTGCTGTACCGTGGTTATGT-3′
*GPX4*	F:5′-GCAGGAGCCAGGAAGTAATC-3′
	R:5′-CCTTGGGCTGGACTTTCAT-3′
*β-actin*	F:5′-GAAGTGTGACGTTGACATCCG-3′
	R:5′-TGCTGATCCACATCTGCTGGA-3′

(*Nrf2*: Nuclear factor E2-related factor 2; *SLC7A11*: Solute carrier family 7 member 11; *GPX4*: Glutathione peroxidase 4).

**Table 2:** Parameters of sperm motility.

Sperm Parameters	Control	ZEA 30	ZEA 30 + Fer−1(0.5)	ZEA 30 + Fer−1(1)	ZEA 30 + Fer−1(1.5)
VSL (μm/s)	40.89 ± 2.35 ^a^	17.56 ± 2.00 ^c^	26.99 ± 1.43 ^b^	24.80 ± 1.22 ^b^	25.66 ± 1.61 ^b^
VCL (μm/s)	90.90 ± 3.63 ^a^	45.17 ± 4.83 ^c^	70.60 ± 2.83 ^b^	70.68 ± 2.30 ^b^	70.99 ± 2.77 ^b^
VAP (μm/s)	47.75 ± 2.47 ^a^	23.11 ± 2.22 ^c^	35.79 ± 1.35 ^b^	32.92 ± 1.38 ^b^	35.21 ± 1.44 ^b^
LIN (%)	44.68 ± 2.21 ^a^	32.53 ± 2.51 ^c^	40.17 ± 1.35 ^b^	40.60 ± 1.05 ^b^	42.02 ± 1.74 ^b^
STR (%)	79.58 ± 1.16 ^a^	72.63 ± 2.56 ^b^	75.26 ± 1.64 ^a^	70.34 ± 1.19 ^b^	75.20 ± 0.85 ^a^
WOB (%)	57.15 ± 2.06 ^a^	50.47 ± 2.52 ^b^	51.40 ± 1.14 ^b^	48.23 ± 0.89 ^b^	51.28 ± 1.72 ^b^

(Values are expressed as mean ± SEM. Different letters within column indicate significant differences (*p* < 0.05). VSL, straight-line velocity; VCL, curvilinear velocity; VAP, average path velocity; LIN, linearity (VSL/VCL); STR, straightness (VSL/VAP); WOB, wobble (VAP/VCL); *n* ≥ 3).

## Data Availability

The data presented in this study are available in the article.

## Supplementary Materials

The following supporting information can be downloaded at: https://www.mdpi.com/article/10.3390/ani12213026/s1, Table S1: CV values of MDA, T-GSH content, SOD activity, Fe^3+^ and Fe^2+^ relative content in each treatment group, Figure S1: Change of diameter of seminiferous tubules in each treatment, Figure S2: Expression changes of *SOX9*, *SYCP3* and *DDX4* proteins in testis of mice in each group, Figure S3: Expression changes of *SLC7A11*, *GPX4* and 4-HNE proteins involved in ferroptosis in testis of mice in each group.
