# Supplementary material for: Effect of Zearalenone-Induced Ferroptosis on Mice Spermatogenesis
Source: Animals (Basel). 2022 Nov 3;12(21):3026. doi: 10.3390/ani12213026 (PMC9657494; doi:10.3390/ani12213026)
Supplement: Supplementary file 1 [file animals-12-03026-s001.zip › animals-1933338-supplementary.pdf]

Table S1. CV values of MDA, T-GSH content, SOD activity, Fe^{3+} and Fe^{2+} relative content in each treatment group.

CV	Control	ZEA 30	ZEA 30+ Fer-1 (0.5)	ZEA 30+ Fer-1 (1)	ZEA 30+ Fer-1 (1.5)
MDA	0.67	0.54	0.28	0.34	0.53
T-GSH	0.15	0.07	0,05	0.06	0.14
SOD	0.08	0.06	0.18	0.07	0.03
Relative content level of Fe^{3+}	0.10	0.08	0.07	0.07	0.15
Relative content level of Fe^{2+}	0.06	0.03	0.03	0.12	0.10

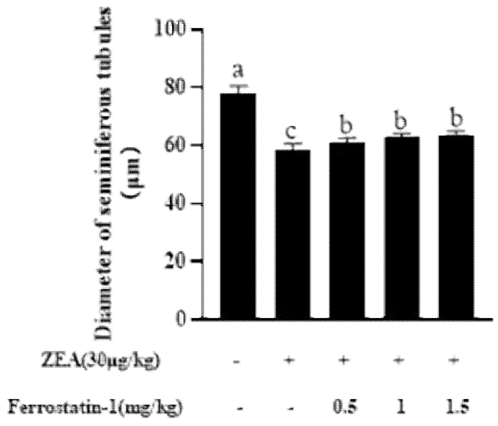

Figure S1. Change of diameter of seminiferous tubules in each treatment.

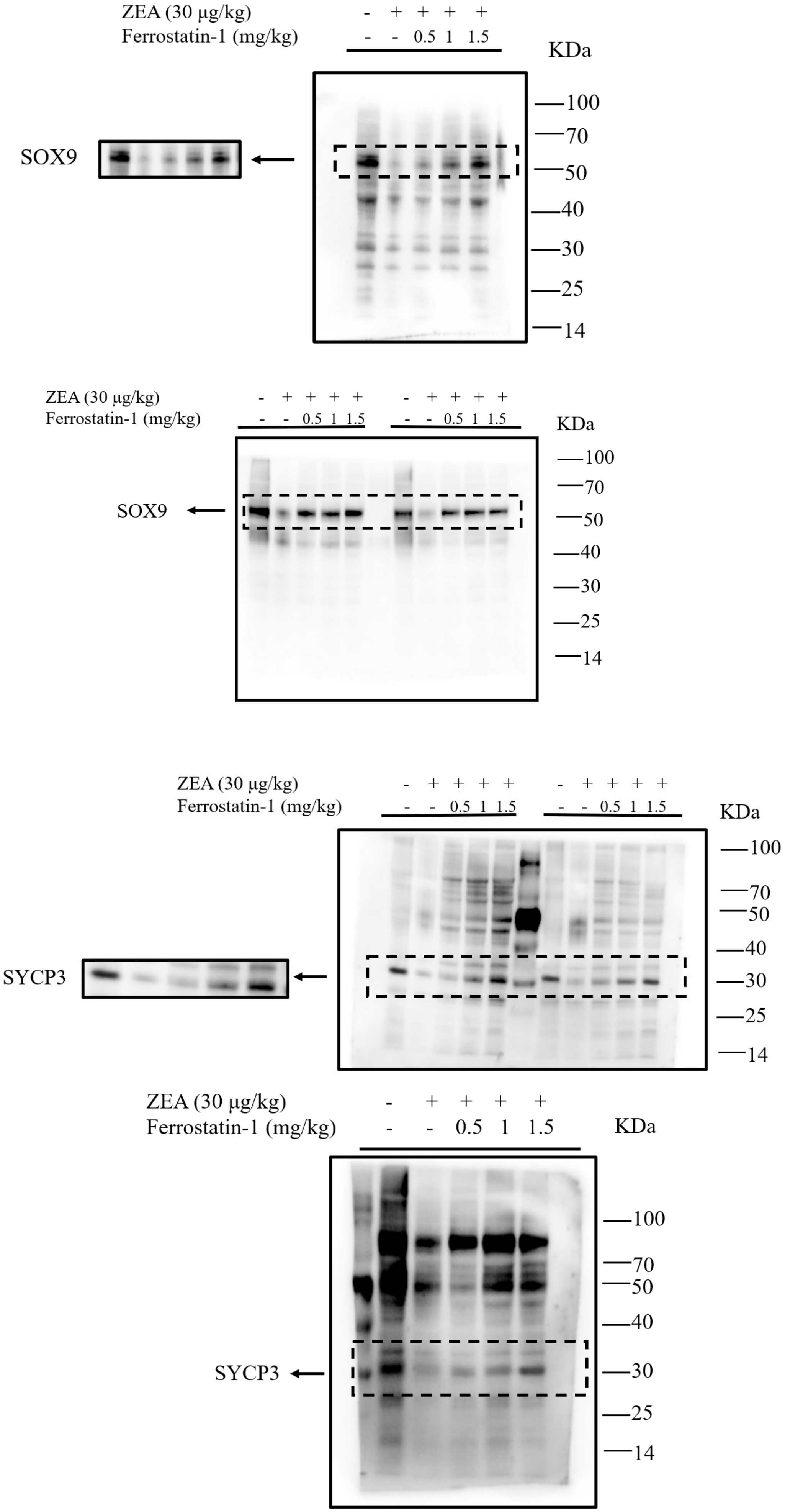
ZEA (30 μg/kg)
Ferrostatin-1 (mg/kg)
- + + + +
- - 0.5 1 1.5
KDa
SOX9
100
70
50
40
30
25
14
ZEA (30 μg/kg)
Ferrostatin-1 (mg/kg)
- + + + + - + + + +
- - 0.5 1 1.5 - - 0.5 1 1.5
KDa
SOX9
100
70
50
40
30
25
14
ZEA (30 μg/kg)
Ferrostatin-1 (mg/kg)
- + + + + - + + + +
- - 0.5 1 1.5 - - 0.5 1 1.5
KDa
SYCP3
100
70
50
40
30
25
14
ZEA (30 μg/kg)
Ferrostatin-1 (mg/kg)
- + + + +
- - 0.5 1 1.5
KDa
SYCP3
100
70
50
40
30
25
14

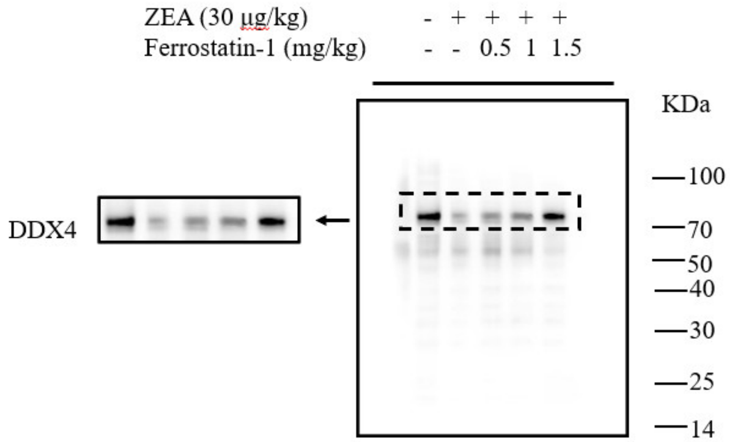
ZEA (30 µg/kg) - + + + +
Ferrostatin-1 (mg/kg) - - 0.5 1 1.5
KDa
DDX4
100
70
50
40
30
25
14

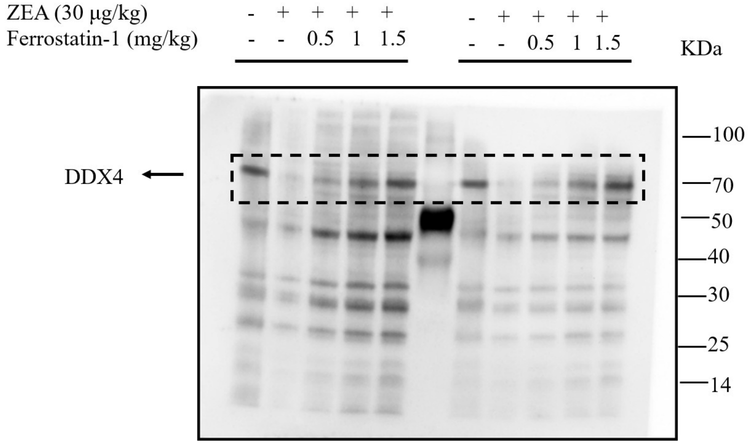
ZEA (30 µg/kg) - + + + + - + + + +
Ferrostatin-1 (mg/kg) - - 0.5 1 1.5 - - 0.5 1 1.5
KDa
DDX4
100
70
50
40
30
25
14

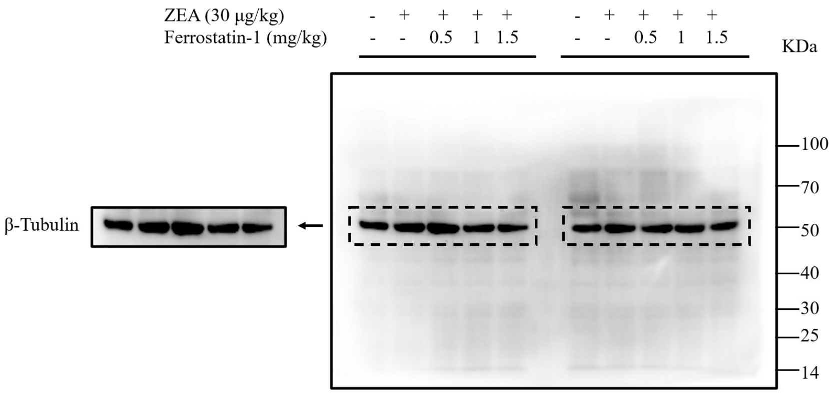
ZEA (30 µg/kg) - + + + + - + + + +
Ferrostatin-1 (mg/kg) - - 0.5 1 1.5 - - 0.5 1 1.5
KDa
β-Tubulin
100
70
50
40
30
25
14

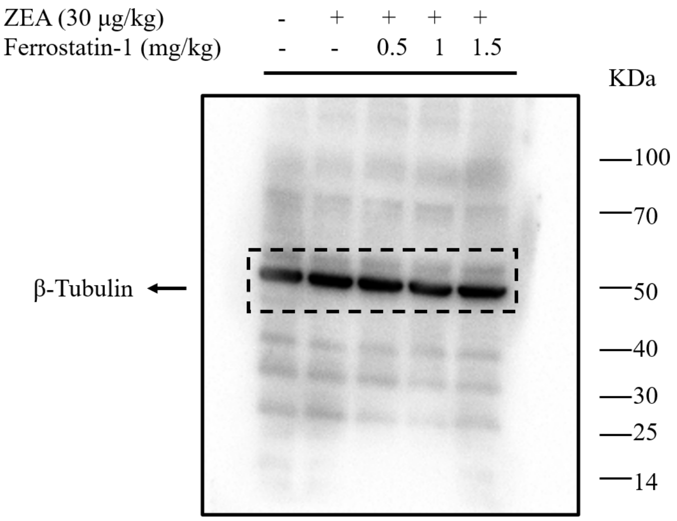
ZEA (30 µg/kg) - + + + +
Ferrostatin-1 (mg/kg) - - 0.5 1 1.5
KDa
β-Tubulin
100
70
50
40
30
25
14

Figure S2. Expression changes of SOX9, SYCP3 and DDX4 proteins in testis of mice in each group.

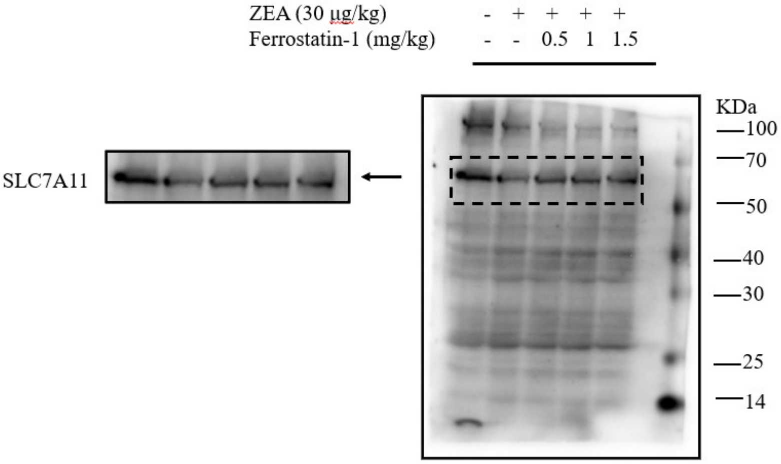

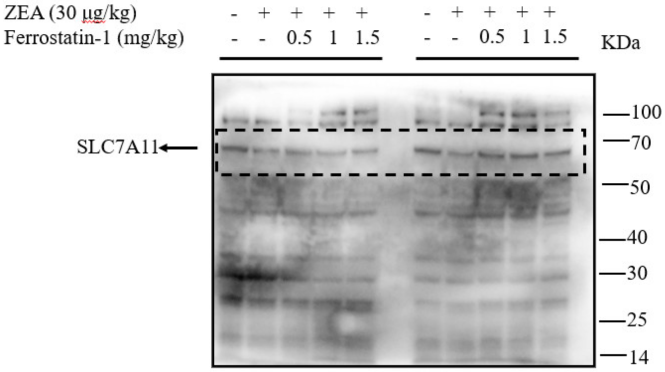

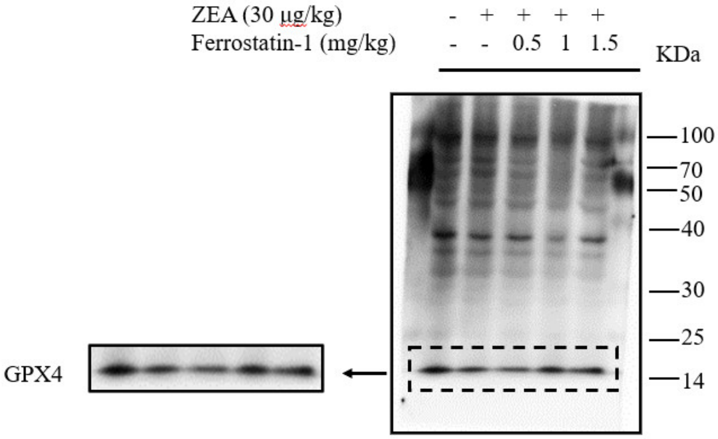

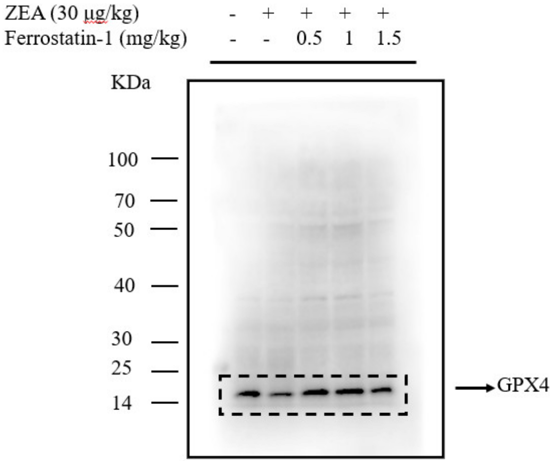

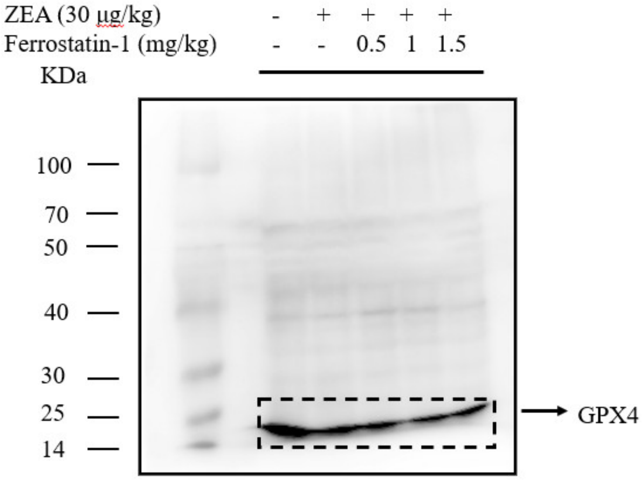

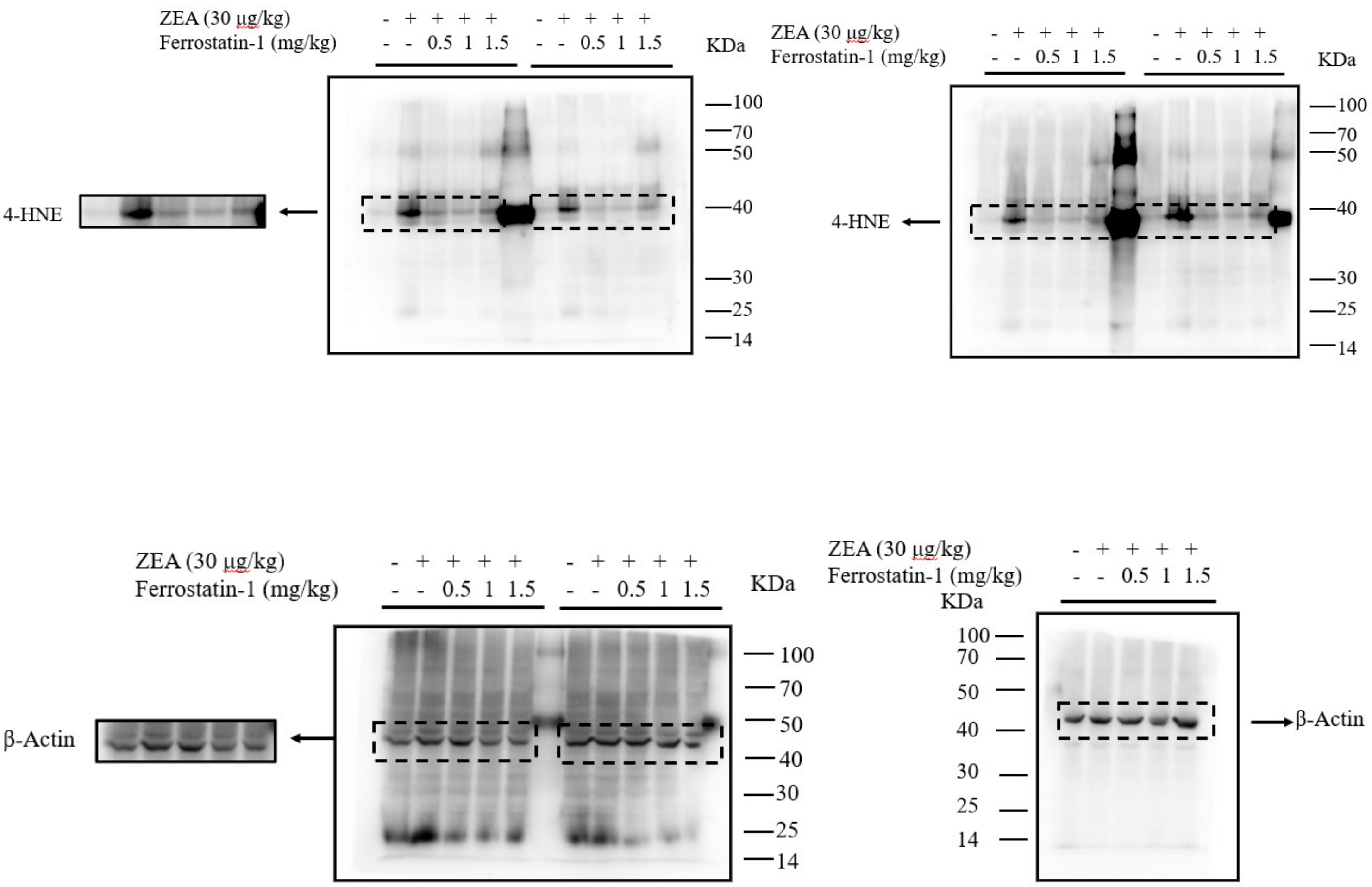

Figure S3. Expression changes of SLC7A11, GPX4 and 4-HNE proteins involved in ferroptosis in testis of mice in each group.
